# Case report: Acute liver failure during deferasirox therapy and the potential role of pharmacogenetics

**DOI:** 10.3389/fphar.2024.1477755

**Published:** 2024-10-23

**Authors:** Belén García-Fariña, Lydia Rink, Virginia Santarini, Marco Westkemper, Christian Dohna-Schwake, Birte Möhlendick

**Affiliations:** ^1^ Institute of Pharmacogenetics, University Hospital Essen, University of Duisburg-Essen, Essen, Germany; ^2^ Department of Pediatrics I, Neonatology, Pediatric Intensive Care, Pediatric Neurology, University Hospital Essen, Essen, Germany; ^3^ Department of Pediatrics III, Pediatric Hematology and Oncology, Cardiology, Pulmonology, University Hospital Essen, Essen, Germany; ^4^ Clinic of Pediatrics, Klinikum Dortmund, Dortmund, Germany

**Keywords:** deferasirox, beta thalassemia, toxicity, ABCC2, ABCG2, UGT1A1, liver failure, pharmacogenetic

## Abstract

**Background and aims:**

A number of case reports have documented the occurrence of acute hepatic and renal toxicity during treatment with deferasirox (DFX). The precise mechanisms underlying these adverse events remain unclear, with the time to toxicity varying considerably between patients—some experiencing it within weeks of treatment initiation, while others after several years. Recent studies have underscored the association of pharmacogenetic variants in genes responsible for the metabolism and clearance of DFX (*ABCC2*, *ABCG2*, and *UGT1A1*) in the development of toxicity. We present the case of an 8-year-old patient with beta thalassemia major who developed acute hepatic failure years after the initiation of DFX therapy. After ruling out the most likely causes, we performed a pharmacogenetic analysis, which suggested a possible link between the patient’s genotype and the development of toxicity.

**Methods:**

Sanger sequencing was performed for the most extensively studied single nucleotide polymorphisms (SNPs) studied associated with changes in transporter/enzyme function: *ABCC2* rs717620 (c.-24C>T), rs2273697 (c.1249G>A), rs8187710 (c.4544G>A), rs369192412 (g.99781071delG); *ABCG2* rs2231142 (c.421C>A); UGT1A1 *6 rs4148323 (c.211G>A), *28 rs3064744 (g.233760235TA[8]), *36 rs3064744 (g.233760235TA[6]) and *37 rs3064744 (g.233760235TA[9]).

**Results:**

The patient is heterozygous for two *ABCC2* variants, namely rs717620 (c.-24C>T) and rs2273697 (c.1249G>A). These variants have the potential to cause a reduction in transporter function, which could in turn result in decreased drug clearance and increased toxicity.

**Discussion:**

The precise mechanism by which toxicity developed in this case remains unclear and is likely multifactorial. However, it is probable that the presence of SNPs in the gene *ABCC2* played a substantial role. Our findings align with those of previously published reports of remarkably similar cases, where patients also exhibited genetic variants in the gene *ABCC2*.

## 1 Introduction

Chelation therapy is an important part of the management of transfusion-dependent patients. Iron chelators are used to prevent and treat iron deposition in end organs by facilitating its elimination and thus preventing its involvement in toxic reactions. Iron accumulation in end organ cells can be potentially cytotoxic through the formation of free radicals, ultimately leading to organ dysfunction. The tissues most commonly affected by iron deposition are the heart, liver, endocrine glands and central nervous system, where it causes a constellation of complications including heart failure and arrhythmias, chronic liver disease, hepatocellular carcinoma, pancreatic dysfunction, diabetes, hypothyroidism, infertility and Alzheimer’s disease (Poggiali et al., 2012; McDowell et al., 2024).

There are currently three iron-specific chelators on the market: deferasirox (DFX), deferiprone (DFP) and deferoxamine (DFO). The three differ in structure, pharmacokinetic properties and adverse event profiles (Table 1).

**TABLE 1:** Comparison of the three chelators commonly used in the treatment of iron overload. According to (Bruin et al., 2008; Waldmeier et al., 2010; Poggiali et al., 2012; Mobarra et al., 2016; Novartis Pharmaceuticals Corporation, 2020; Novartis Pharmaceuticals Corporation, 2022; European Medicines Agency, 2024).

	Deferasirox (DFX)	Deferiprone (DFP)	Deferoxamine (DFO)
Route of administration	Oral	Oral	Subcutaneous or intravenous
Half-life	8–16 h	3-4 h	20–30 min
Pharmacokinetics	Metabolized through UGT1A1 and UGT1A3 enzymes. Marginal (<8%) oxidative metabolism through CYP450 enzymes Eliminated through feces and to a lesser extent (<8%) in urine	Metabolized by UGT1A6 enzymes 90% excreted in urine	Metabolism through plasma enzymes (pathways not yet defined) Urinary excretion with small percentage in feces
Frequency of dosing	Once daily	Three times daily	8–24 h long continuous infusion, 5–7 days per week
Adverse effects	Cutaneous rash, gastrointestinal disturbances, increase in serum creatinine, Fanconi syndrome, renal and liver failure	Agranulocytosis, neutropenia, and arthralgia	Cutaneous reactions, visual and auditory neurotoxicity, hypotension
Advantages	Improves compliance	Effective in cardiac iron elimination	High molar iron chelating efficiency (hexadentate complex)
Challenges	Monitoring of renal and hepatic function	Blood count monitoring Variable efficacy in removing hepatic iron	Adherence Frequent ophthalmologic and auditory examinations

**FIGURE 1:**
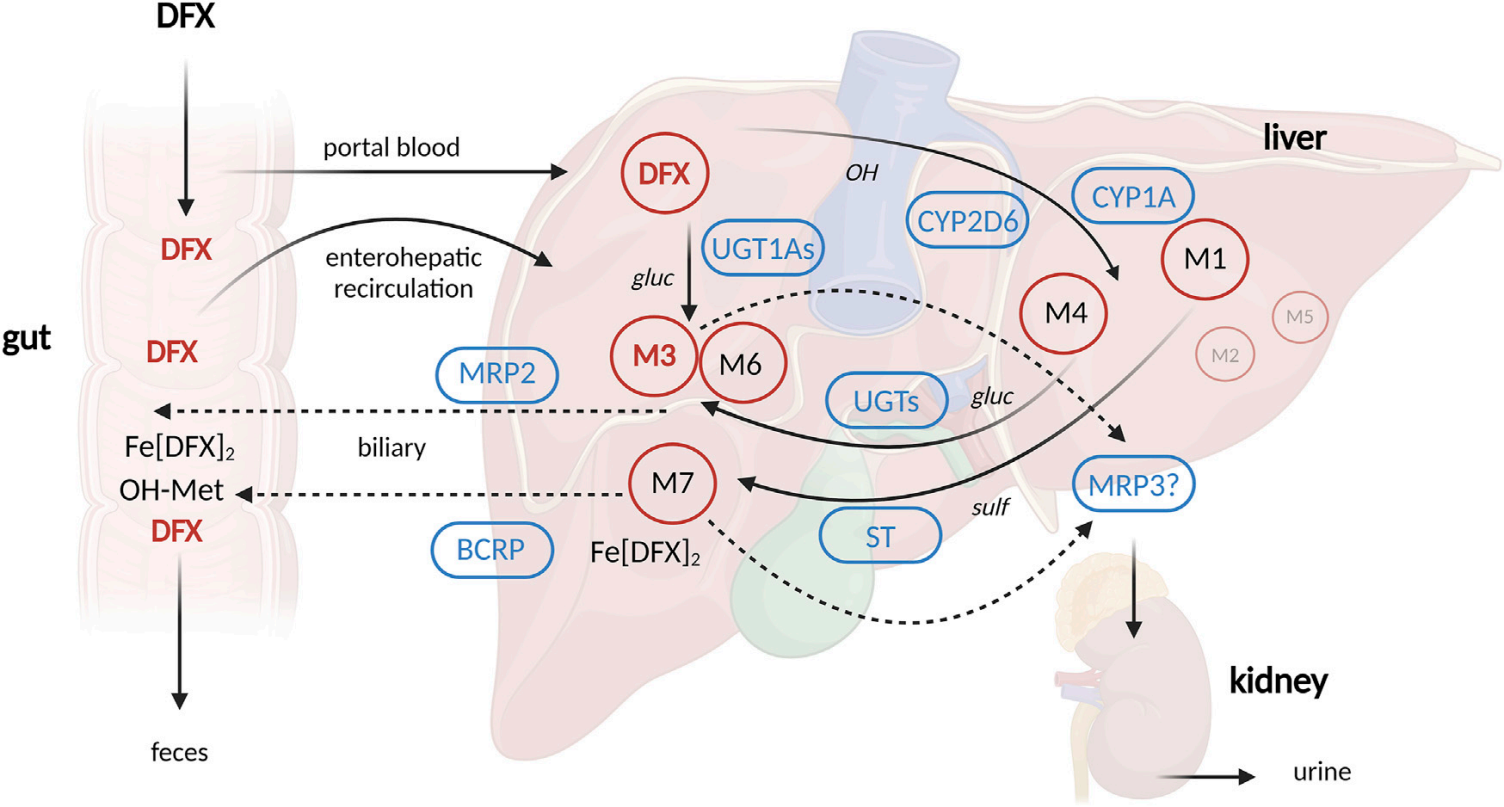
Schematic depiction of deferasirox pharmacokinetics. Created with Biorender.com (publication license OO277FX3OK) according to Waldmeier et al., 2010; Waldmeier et al., 2010). Abbreviations: BCRP = breast cancer resistance protein, CYP = cytochrome P450, DFX = deferasirox, Fe [DFX]_2_ = DFX-Iron complex, M = metabolite (M1, M4, M7 = hydroxylated metabolites, M3, M6 = glucuronides), MRP2 = Multidrug resistance-associated protein 2, MRP3 = Multidrug resistance-associated protein 3, ST = sulfotransferase, UGT1A1 = UDP glucuronosyltransferase 1 family, polypeptide A1, gluc = O-glucuronidation, OH = hydroxylation, sulf = O-sulfation.

DFX is a tridentate iron chelator that revolutionized the market in 2005 as the first oral, once-daily agent of its kind (Mobarra et al., 2016; Entezari et al., 2022). As a result, DFX is predominantly used in pediatrics. After absorption in the gastrointestinal tract, DFX is metabolized in the liver mainly by glucuronidation by uridine diphosphate glucuronosyltransferases (UGT), mainly UGT1A1 and to a lesser extent UGT1A3. A small percentage (6%–8%) is oxidized by cytochrome P450 (CYP) 1A1 and 2D6. The resulting metabolites are then excreted into the bile by cellular transporters of the ATP-binding cassette (ABC) superfamily, in particular MRP2 (multidrug resistant protein 2) and BCRP (breast cancer-related protein), and eliminated in the feces. A small percentage (∼8%) is excreted in the urine, and multidrug resistant protein 3 (MRP3) is thought to be responsible for its transport into the sinusoidal blood stream (Bruin et al., 2008; Waldmeier et al., 2010) (Figure 1).

The side effects of DFX are well documented. The most common adverse reactions are elevations in serum creatinine (occurring in ≥1/10 patients), rash, gastrointestinal symptoms and elevation in transaminases (≥100 to <1/10 patients). Side effects like hepatitis and acquired Fanconi syndrome are considered rare (occurring in ≥1/10.000 to <1/1.000 patients). Overt hepatic and renal failure have been reported but the frequency of those events cannot be estimated from the available data (Novartis Pharmaceuticals Corporation, 2020).

Recent literature has highlighted the association between DFX toxicity and pharmacogenetic variations (Choi et al., 2007; Lee et al., 2013; Cusato et al., 2015; Allegra et al., 2016; Wen et al., 2017; Allegra et al., 2018). This association appears to be related to genetic polymorphisms in the genes responsible for DFX metabolism and disposition, specifically *UGT1A1*, *ABCC2* (encoding MRP2), and *ABCG2* (encoding BCRP). Certain genotypes have been shown to affect cellular transport activity, resulting in decreased drug clearance and increased toxicity.

In a recent case at our university hospital, an 8-year-old boy with beta thalassemia major treated with DFX was admitted to the pediatric intensive care unit with acute liver failure and hyperammonemia. After excluding the most likely causes (infection, intoxication, inborn errors of metabolism, etc.), our pharmacogenetic department was consulted for further diagnostic insight.

In the following report, we discuss the potential use of pharmacogenetic testing as a predictive tool for DFX toxicity.

## 2 Case description

Our case focuses on an 8-year-old boy of Syrian descent who was diagnosed with beta thalassemia major at the age of 6 months. Since his diagnosis in 2016, he has received regular blood transfusions. In April 2017, he was started on chelation therapy with DFX (250 mg/day) due to an elevated ferritin level (>1,000 μg/L). The patient continued to receive transfusions every 3-4 weeks without complications and maintained a satisfactory ferritin level. At the time of the events, his daily DFX dose was 360 mg/day (18.9 mg/kg) and was not receiving any other pharmacologic treatment. Regarding family history, the parents of the child reported to be consanguineous and the patient has two siblings who do not require transfusions. However, we do not have information on whether the siblings have been tested for beta thalassemia.

In October 2023, 2 weeks after his last red blood cell transfusion, the child developed a gastrointestinal infection with high fever (39.9°C) and vomiting, which was treated with oral dimenhydrinate.

Within 12 h of starting symptomatic treatment, the patient’s condition began to deteriorate. He was admitted to his local hospital, where he presented with markedly decreased general condition, cyanosis, hypothermia, and deep somnolence, as well as increased muscle tone and perioral and ocular spasms.

Initial blood gas analysis (BGA) and laboratory tests revealed metabolic acidosis and profound hypoglycemia (27.0 mg/dL), mild lactate acidosis (2.50 mmol/L), and severe hyperammonemia (509.0 μmol/L). Liver enzymes were mildly elevated (ALT 84.0 U/L, AST 100.0 U/L, AP 364.0 U/L), as were uric acid (45.0 mg/dL) and calcium (3.05 mmol/L), thrombocytopenia (172,000/µL) and elevated INR (1.98).

Because of clinical suspicion of encephalitis, intravenous ceftriaxone, acyclovir and dexamethasone were given empirically and later discontinued because urine, blood, cerebrospinal fluid cultures, and viral serology were all negative and a respiratory virus panel PCR was positive for rhinovirus.

An initial cranial MRI showed no pathology and the EEG displayed generalized slowing without evidence of epileptic foci.

Within 24 h of hospital admission, the child was intubated due to the absence of protective reflexes and notable decline of alertness, and was subsequently transferred to our intensive care unit for further treatment. Continuous venovenous hemodialysis (CVVHD) was initiated, resulting in a notable reduction in ammonia level (70 mmol/L) within 48 h.

Given the high probability of a urea cycle defect, a sodium benzoate infusion was initiated but discontinued upon the exclusion of inborn errors of metabolism through genetic testing. Additionally, a continuous levocarnitine infusion was administered due to a markedly reduced free carnitine level, which could be secondary to the patient`s initial decline or a primary disorder of the carnitine transporter. However, this is not typically associated with hyperammonemia, and genetic testing also ruled out this possibility.

The child’s condition showed gradual improvement and he was discharged 1 week later in good general condition. At the time of admission, chelation with DFX was withheld due to the known potential hepatic side effects. Since then, he has continued to receive regular erythrocyte transfusions and the iron chelation has been changed to DFP 1000 mg/day (46.1 mg/kg/day). His renal and hepatic function have been closely monitored and have remained stable since that time.

A pharmacogenetic analysis was conducted in our institute while the patient was still in the ICU department of our university hospital; however, the results did not influence the subsequent treatment decisions. The child’s parents provided written informed consent. A venous blood sample was collected in an EDTA tube 1 month after the patients’ last transfusion to confirm that the venous blood from the patient was not contaminated with donor blood. DNA extraction was conducted using the QIAamp DNA Blood kit (Qiagen, Hilden, Germany) according to the manufacturer’s instructions. Sanger sequencing was performed for the most extensively studied single nucleotide polymorphisms (SNPs) that have been associated with changes in transporter/enzyme function (Choi et al., 2007; Bruin et al., 2008; Lee et al., 2013; Cusato et al., 2015; Allegra et al., 2016; Wen et al., 2017): *ABCC2* rs717620 (c.-24C>T), rs2273697 (c.1249G>A), rs8187710 (c.4544G>A), rs369192412 (g.99781071delG); *ABCG2* rs2231142 (c.421C>A); UGT1A1 *6 rs4148323 (c.211G>A), *28 rs3064744 (g.233760235TA[8]), *36 rs3064744 (g.233760235TA[6]) and *37 rs3064744 (g.233760235TA[9]).

Information regarding covered genomic regions and polymerase chain reaction (PCR) conditions can be found in Supplementary Table S1. Briefly, PCR was performed using 2 µL of genomic DNA and 30 µL Taq DNA-Polymerase 2x Master Mix Red (Ampliqon, Odense, Denmark) with the following conditions: initial denaturation at 95°C for 5 min; 35 cycles of denaturation at 95°C for 30 s, annealing at the respective Tm (given in Supplementary Table S1) for 30 s, and extension at 72°C for 30 s each; final extension at 72°C for 10 min. PCR products were prepared for Sanger sequencing according to manufacturer’s instructions: In brief, 5 µL PCR product was mixed with 2 µL ExS-Pure™ Enzymatic PCR Cleanup Kit (NimaGen, Nijmegen, Netherlands), vortexed and incubated for 4 min at 37°C, followed by heat inactivation for 1 min at 90°C. Next, cycle sequencing was performed using the Brilliant Dye^®^ Terminator 3.1 Sequencing Kit (NimaGen, Nijmegen, Netherlands) with the following thermal cycling protocol: 96°C for 1 min; 25 cycles at 96°C for 10 s, 50°C for 5 s and 60°C for 2 min. Thereafter, the cycle sequencing reaction was purified using the iX-Pure™ Dye Terminator Cleanup Kit (NimaGen, Nijmegen, Netherlands). Sanger sequencing was performed on the SeqStudio™ genetic analyzer system (Thermo Fisher Scientific, Darmstadt, Germany).

The patient is heterozygous for two *ABCC2* variants: rs717620 (c.-24C>T) and rs2273697 (c.1249G>A) (Table 2). All other tested variants were found to be homozygous reference genotype. Based on the available evidence, it is reasonable to suggest that the observed genetic variations may be a contributing factor in the development of toxicity in our patient.

**TABLE 2:** Pharmacogenetic analyses and results.

Gene	SNP	Position^a^	Protein	Genotype	Functional effect
*UGT1A1*	*6 rs4148323	NM_000463.3: c.211G>A	NP_000454.1: p.Gly71Arg	GG	Reduced glucuronidation
	*1 rs3064744	NC_00002.12: g.233760235TA[7] / A(TA)_6_TAA	-	TA [7]TA [7]	Normal function
	*28 rs3064744	NC_00002.12: g.233760235TA[8] / A(TA)_7_TAA			Reduced glucuronidation
	*36 rs3064744	NC_00002.12: g.233760235TA[6] / A(TA)_5_TAA			Increased glucuronidation
	*37 rs3064744	NC_00002.12: g.233760235TA[9] / A(TA)_8_TAA			Reduced glucuronidation
*ABCC2*	rs369192412	NC_011798.2: g.99781071delG	-	GG	Reduced transport
	rs717620	NM_000392.5: c.-24C>T	-	CT	Reduced transport
	rs2273697	NM_000392.5: c.1249G>A	NP_000383.1: p.Val417Ile	GA	Reduced transport
	rs8187710	NM_000392.5: c.4544G>A	NP_000383.1: p.Cys1515Tyr	GG	Reduced transport
*ABCG2*	rs2231142	NM_004827.3: c.421C>A	NP_004818.2: p.Gln141Lys	CC	Reduced transport

^a^ Position according to HGVS (Human Genome Variation Society) Nomenclature and UGT Nomenclature Committee (https://www.pharmacogenomics.pha.ulaval.ca/wp-content/uploads/2015/04/UGT1A1-allele-nomenclature.html).

## 3 Discussion

Given that elevations in serum creatinine and transaminases are reported at a relatively high incidence during treatment (≥1/10 patients and ≥100 to <1/10 patients respectively), DFX carries a boxed warning for hepatic and renal toxicity, which requires assessment of renal and hepatic function prior to initiation of treatment and at least monthly thereafter (Novartis Pharmaceuticals Corporation, 2020).

The precise mechanisms underlying DFX-induced hepatic and renal toxicity remain unclear. The onset of adverse effects occurs between days and several years after the initiation of therapy and is typically reversible following the discontinuation of the drug (National Institute of Diabetes and Digestive and Kidney Diseases, 2012; Díaz-García et al., 2014). A research group investigating the nephrotoxic effects of DFX discovered that the drug could cause direct sublethal or lethal tubular cell injury in animal studies (Díaz-García et al., 2014). Additionally, a Swiss research group proposed that mitochondrial swelling may be the underlying cause of DFX toxicity (Gottwald et al., 2020).

It is reasonable to propose that genetic polymorphisms causing suboptimal drug elimination and consequent increased drug exposure may lead to a higher incidence of adverse effects, irrespective of the molecular mechanism behind the development of toxicity. Nevertheless, the observed delay in the onset of adverse effects cannot be fully attributed to genetic changes. This suggests that, while pharmacogenetic variations may contribute to the development of severe toxicity, the underlying etiology is likely to be multifactorial.

In recent years, several studies have described that SNPs, particularly in the genes encoding the cellular transporters involved in DFX disposition, may increase susceptibility to DFX toxicity (Choi et al., 2007; Lee et al., 2013; Cusato et al., 2016; Allegra et al., 2017; Wen et al., 2017).

The effect of genetic variations in the *ABCC2* gene (encoding the MRP2 protein) has been the subject of extensive investigation yielding consistent results that suggest a potential association between variants and the occurrence of complications due to inefficient drug elimination (Choi et al., 2007; Lee et al., 2013; Allegra et al., 2016; Cusato et al., 2016; Wen et al., 2017).

A study conducted on a Korean cohort revealed that a computationally defined haplotype comprising the *ABCC2* rs717620 (c.-24C>T) SNP together with rs1885301 (g.3699A>G), rs2804400 (c.334-178 49C>T) and rs3740066 (c.3972C>T) was found at a significantly higher frequency (*P* = 0.02) in the group of patients who developed toxic hepatitis compared to the controls. Moreover, through a luciferase-based reporter assay performed in HepG2 cells, the same haplotype exhibited a 40% reduction in basal promoter activity, suggesting diminished transporter expression and consequently reduced drug elimination (Choi et al., 2007).

An Italian research group has extensively studied the association of SNPs in genes relevant for DFX pharmacokinetics. These genes include *UGT1A1* rs887829 (g.233759924C>T), *UGT1A3* rs3806596 (c.-66T>C) and rs1983023 (g.233728376T>C), *CYP1A1* rs2606345 (c.-27+606G>T) and rs4646903 (g.74719300A>G), CYP1A2 rs762551 (c.-9-154C>A) and rs2470890 (c.1548T>C), CYP2D6 rs1135840 (c.1457G>C), ABCC2 rs2273697 (c.1249G>A), ABCG2 rs2231142 (c.421C>A) and rs13120400 (c.1194+928A>C). They found that carriers of the A-allele in ABCC2 rs2273697 (c.1249G>A) had a higher DFX plasma area under the curve (AUC) and plasma exposure at the end of the dosing interval (C_trough_), which supports the hypothesis of a lower transport activity in A-allele carriers (Cusato et al., 2015; Cusato et al., 2016; Allegra et al., 2017). These findings are in line with a study published in 2017, which concluded through functional studies (*in vitro* quantification of cell surface transport expression) that *ABCC2* rs2273697 (c.1249G>A) has reduced transport activity (Wen et al., 2017).

In 2013, pharmacogenetic assessment was performed in 98 Korean pediatric patients receiving DFX for the treatment of iron overload with various hematological conditions. The results demonstrated that the patients carrying two computationally determined ABCC2 haplotypes, each containing rs717620 (c.-24C>T) and/or rs369192412 (g.99781071delG), had an increased risk of hepatotoxicity compared to the wild-type allele. Of the entire patient cohort, 15 individuals developed hepatotoxicity, of which six were heterozygous (out of the 38 heterozygous patients) and two homozygous (out of two mutant homozygous patients in the cohort) for *ABCC2* rs717620 (c.-24C>T) (Lee et al., 2013).

Several published case reports have highlighted adverse reactions to DFX, with patients commonly presenting with symptoms of acute liver failure, Fanconi syndrome of varying severity, or a combination of both (Rafat et al., 2009; Grangé et al., 2010; Papadopoulos et al., 2010; Yacobovich et al., 2010; Rheault et al., 2011; Wei et al., 2011; Ling et al., 2015; Braga et al., 2017; Menaker et al., 2017; Ramaswami et al., 2017; Martinelli et al., 2019).

A review of the literature reveals that pharmacogenetic analysis has only been considered in two of the published case reports. The initial case report involved a three-year-old girl with beta thalassemia major who developed acute liver failure and Fanconi syndrome, a case remarkably similar to that of our patient. The patient had started chelation therapy with DFX the previous year and in the months following, she began to show signs of toxicity (fever, vomiting, azotemia) eventually developing overt liver failure and hyperammonemia (Marano et al., 2016). The patient was found to be heterozygous for UGT1A1*60 rs4124874 (g.233757013T>G), *ABCC2* rs717620 (c.-24C>T) and *ABCC2* rs8187710 (c.4544G>A). The second case, a 43-year-old woman with sickle cell disease who died of fulminant liver failure 56 days after starting DFX, tested homozygous for *ABCC2* rs369192412 (g.99781071delG). This patient had no history of hepatic sequestration, was not taking any potentially hepatotoxic medications and did not consume alcohol. Two months after the initiation of treatment, her transaminases level increased 4-fold the upper limit of normal (ULN) and DFX was discontinued. Despite this, the liver failure continued to evolve and she passed away 26 days after admission (Braga et al., 2017).

The precise etiology of the severe toxicity observed in our patient remains uncertain. A number of factors may have been involved, including DFX, the infection, and potentially the initial dimenhydrinate therapy (although no interactions between these two drugs have been described, and they have different pharmacokinetic profiles). Furthermore, the rationale behind the emergence of these severe adverse effects following an extended period of DFX treatment is yet not clear. A review of the literature reveals that patients have experienced adverse effects at various points during the course of treatment, spanning from the early stages of therapy, within days of its commencement, to instances occurring years later.

Based on the existing literature and the genotype of our patient, heterozygous for both *ABCC2* rs717620 (c.-24C>T) and *ABCC2* rs2273697 (c.1249G>A), it seems plausible to suggest that the observed toxicity is related to pharmacogenetic variations in the cellular transporters involved in the hepatic clearance of DFX. Furthermore, it is noteworthy that the child’s hepatic and renal functions have remained stable since the start of treatment with deferiprone (DFP). This consistency could be explained by the different pharmacokinetic profiles of DFX and DFP, with DFP having minimal biliary elimination.

Our findings align with studies linking specific genetic variations in relevant cellular transporters to the development of toxicity in patients undergoing DFX therapy. While further research with larger cohorts is warranted to generalize recommendations, the consistency of our observations with existing research suggests that pharmacogenetic testing for *ABCC2* polymorphisms could play a potential role in clarifying the etiology of toxicity in DFX therapy. This approach could contribute to a more personalized and safer treatment strategy. Additionally, clinicians should be encouraged to remain informed about the influence of pharmacogenetic factors in the development of toxicity during DFX treatment.

## Data Availability

The original contributions presented in the study are included in the article/Supplementary Material, further inquiries can be directed to the corresponding author.
