# Supplementary material for: Case report: Acute liver failure during deferasirox therapy and the potential role of pharmacogenetics
Source: Front Pharmacol. 2024 Oct 23;15:1477755. doi: 10.3389/fphar.2024.1477755 (PMC11538451; doi:10.3389/fphar.2024.1477755)
Supplement: Supplementary file 1 [file DataSheet1.PDF]

Supplementary Material

Supplementary Table 1. Genomic localization and population genetics of the investigated pharamacogenes.

Gene	SNP	Position[#]	Genomic regions covered (length bp)	Tm	Minor allele frequencies (gnomAD v4.1.0)				
					NFE	MID	AFR	SAS	EAS
UGT1A1	**6* rs4148323	NM_000463.3: c.211G>A	Chr2:233760362-233760690 (329 bp)	60 °C	0.001	0.002	0.001	0.019	0.164
	**28* rs3064744	NC_00002.12: g.233760235TA[8] / A(TA)$_7$TAA	Chr2:233760017-233760476 (419 bp)	55 °C	0.306	0.3146	0.375	0.361	0.121
	**36* rs3064744	NC_00002.12: g.233760235TA[6] / A(TA)$_5$TAA	Chr2:233760017-233760476 (419 bp)		0.001	0.003	0.050	0.002	0.0002
	**37* rs3064744	NC_00002.12: g.233760235TA[9] / A(TA)$_8$TAA	Chr2:233760017-233760476 (419 bp)		0.003	0.004	0.077	0.001	0.001
ABCC2	rs369192412	NC_000010.11: g.99781071delG	Chr10:99780819-99781446 (628 bp)	57°C	0.034	0.082	0.066	0.324	0.434
	rs717620	NM_000392.5: c.-24C>T	Chr10:99782672-99782975 (304 bp)	57°C	0.202	0.132	0.058	0.112	0.205
	rs2273697	NM_000392.5: c.1249G>A	Chr10:99803885-99804204 (320 bp)	57°C	0.201	0.261	0.184	0.277	0.106
	rs8187710	NM_000392.5: c.4544G>A	Chr10:99851206-99851603 (398 bp)	60°C	0.057	0.134	0.160	0.019	0.0004
ABCG2	rs2231142	NM_004827.3: c.421C>A	Chr4:88130921-88131222 (302 bp)	57°C	0.111	0.057	0.027	0.090	0.298

Abbreviations: Chr = Chromosome; bp = base pairs; Tm = Primer melting temperature; NFE = Non-Finnish European; MID = Middle Eastern; AFR = African; SAS = South Asian; EAS = East Asian. [#]Position according to HGVS (Human Genome Variation Society) Nomenclature and UGT Nomenclature Committee (https://www.pharmacogenomics.pha.ulaval.ca/wp-content/uploads/2015/04/UGT1A1-allele-nomenclature.html).
